# A Transdiagnostic Approach to Sexual Distress and Sexual Pleasure: A Preliminary Mediation Study with Repetitive Negative Thinking

**DOI:** 10.3390/ijerph17217864

**Published:** 2020-10-27

**Authors:** Patrícia M. Pascoal, Catarina F. Raposo, Magda Sofia Roberto

**Affiliations:** 1 CICPSI, Faculdade de Psicologia, Universidade de Lisboa, 1649-013 Lisboa, Portugal; msroberto@psicologia.ulisboa.pt; 2 Escola de Psicologia e Ciências da Vida, Universidade Lusófona de Humanidades e Tecnologias, 1649-013 Lisboa, Portugal; 3 CPUP, Faculdade de Psicologia e Ciências da Educação, Universidade do Porto, Rua Alfredo Allen, 4200-135 Porto, Portugal; catarinajraposo@gmail.com

**Keywords:** sexual distress, sexual pleasure, transdiagnostic factors, perseverative cognitions, repetitive negative thinking, worry, rumination, co-worry, co-rumination

## Abstract

Sexual distress is a core characteristic of sexual dysfunction; however, little is known about its correlates. In the current study, we aimed to contribute to the understanding of both sexual distress and its positive counterpart, sexual pleasure, by taking a transdiagnostic approach to sexual distress using two types of repetitive negative thinking: worry and rumination. Because sexual activity mostly occurs in a dyadic context, we also looked at the potential mediating effect of co-worry and co-rumination, and we used them as mediators. Our preliminary exploratory quantitative study used a cross-sectional design, with a sample of 206 partnered heterosexual people. We used path analysis with parallel mediation, with structural equation modelling being performed using lavaan designed for R environment. Overall, our results show that repetitive negative thinking is associated with both sexual distress and sexual pleasure, and that neither co-rumination nor co-worry mediates these associations. The exception is the indirect effect of rumination on sexual pleasure that is mediated by co-rumination. These results demonstrate that a transdiagnostic approach to sexual distress is a new field worth exploring, and they contribute to establishing the relevance of a cognitive approach to sexual dysfunction.

## 1. Introduction

Mental health studies have been characterised by a shift from a categorical to a dimensional approach. This change has resulted from empirical evidence that has clearly shown comorbidity in diagnoses to be the rule, not the exception. Such evidence has also demonstrated that a categorical approach, based on the dichotomy of existence or absence of a diagnosis, fails to grasp the underlying dimensions of some mental disorders [1]. These underlying dimensions are processes [2] that are common to different mental health problems and include perseverative cognitions, or repetitive negative thinking, such as rumination and worry [3]. Research has demonstrated that these underlying dimensions frequently explain high levels of personal distress that are experienced.

Personal distress is a necessary condition for the diagnosis of most mental health problems. Therefore, for a diagnosis to be made, it is required that there should be not only the presentation of a set of signs and symptoms, but also that person’s experience of personal distress to accompany these signs and symptoms. Sexual dysfunctions (e.g., erectile disorder, premature ejaculation, penetration/pain disorder and others) constitute a set of sexual functioning problems whose core characteristic is the experience of sexual distress alongside sexual functioning. So, according to a transdiagnostic approach, transdiagnostic factors (such as worry and rumination) are usually associated with high levels of emotional distress in relation to most common mental health problems (such as depression and anxiety). It is likely that these factors that are associated with emotional distress are associated with sexual distress. Findings on comorbidity relating to depression, anxiety and sexual dysfunction support this possibility [4,5]. However, to the best of the authors’ knowledge, there have so far been no studies to show that a transdiagnostic approach is suitable for understanding sexual distress with sexual functioning.

Although worry and rumination share some common characteristics, such as uncontrollable repetitive thinking, which explain their strong association, there are also some differences between these two activities. Worry is characterised by abstract thinking and a chain of thoughts and images that reflect speculation about the uncertain outcome of future events [6]. Worry can be adaptive and have beneficial effects (such as forming effective problem-solving strategies for future threats) and is linked to preventive and protective behaviours such as health surveillance and seatbelt use [7]. However, high levels of worry, or worry about unlikely events, lead to worry behaviours and are associated with negative mental health results such as generalised anxiety disorder [8]. While some studies have associated worry with sexual outcomes (e.g., erectile disorder; low sexual desire) [9,10], no study that focuses on mental-health-related factors, namely transdiagnostic factors, was found that associated worry as a pattern of thinking with sexual distress or diminished sexual pleasure, and it is logical for the current study to fill this gap.

Rumination is a process of negative thinking that focuses on past or present negative emotional experiences and is characterised by a dwelling upon self-worth, loss and failure, related to specific events [11]. Rumination is strongly associated with negative mental health outcomes such as depression and anxiety [12]. In studies of sexuality, rumination has been studied mainly in the context of its association with experiences of sexual abuse, trauma or stigmatisation [13,14]. No studies were found that explored a possible association between this transdiagnostic factor and sexual distress or pleasure, and this is another research gap that the current study aims to fill.

Sexual functioning most commonly occurs in a dyadic context. According to interdependence theory, individuals influence one another’s experiences (i.e., their thoughts, emotions, motives, behaviour and outcomes) through their interactions. According to this theory, amorous dyads might be expected to be characterised by interdependence and, subsequently, relationship factors and processes will have a role in the quality of sexual response [15]. Research has demonstrated that a partner’s sexual problem affects both members of the couple [16]. Moreover, having a partner seems to be an important factor in explaining the presence of distress in women with low sexual desire and is an important factor that shapes men’s sexual desire, which implies that sexual desire problems are markedly interpersonal and highlights the importance of studying interpersonal factors [17,18]. Further research has demonstrated that partner issues are associated with the experience of distress in relation to other dimensions of sexual function (e.g., orgasm) [19,20].

An interpersonal transdiagnostic factor that may have a role in sexual distress with sexual functioning may be co-rumination, a well-established factor involved in emotional distress in friendship dyads [21]. Co-rumination is characterised by repeatedly talking about, and sharing, problems with a peer, and differs from self-disclosure in that it focuses on speculating about, discussing and revisiting a problem. Co-rumination has been studied mainly with samples of children, adolescents and non-romantic couples [22,23,24]. The research on friendship dyads shows that co-rumination is related to the process of interpersonal adaptation, specifically adaptation in friendly relationships (e.g., by promoting the perception of greater self-disclosure, closeness, intimacy, security, acceptance and validation in the friendship relationship) [24]. Co-rumination is also associated with difficulties in the process of intrapersonal adaptation, particularly with emotional adaptation (e.g., experiencing low self-esteem, internalising symptoms) [22]. Due to its important role as a maintaining factor for emotional distress, co-rumination about a sexual problem could be involved in the experience of sexual distress related to that problem.

Another possible interpersonal factor is co-worry, which has also been present in studies of emotional adjustment with samples of adolescents and friendship dyads [21]. Co-worry can be a feature of interpersonal behaviour, which can be triggered by one’s worry thinking style [25]. It is a dyadic conversation style for concerns about a perceived threat to be shared and discussed repeatedly. The threat is interpreted and evaluated, the negative future event is projected and there is a perception of the inability to control the worry, as well as some effectiveness in developing coping strategies to fight it. Co-worry may be a warning signal in a relationship about possible dangers (an alerting function) or an attempt to seek interpersonal comfort or assistance (a comfort-seeking function) [26,27]. This definition is similar to that of co-rumination, but it differs in that it relates to an anxious pattern of communication in the relationship [23,28]. The interpersonal counterparts of worry and rumination, i.e., co-worry and co-rumination, have been overlooked in sexual-response-related studies. In the current study, co-rumination and co-worry are considered to be potential mediators between repetitive negative thinking and sexual distress and sexual pleasure.

Although the study of transdiagnostic factors in sexual dysfunction seems a logical conceptual step, research devoted to transdiagnostic factors in relation to sexuality has focused mainly on the mental health of sexual minorities [29] or sexually aggressive behaviour [30]. It is necessary to clarify whether transdiagnostic factors associated with the most common comorbidities of sexual dysfunction (anxiety and depression) are also associated with sexual distress about sexual functioning. The current exploratory preliminary cross-sectional study aimed to clarify this possibility using a sample of cisgender people involved in a monogamous relationship.

**Figure 1:**
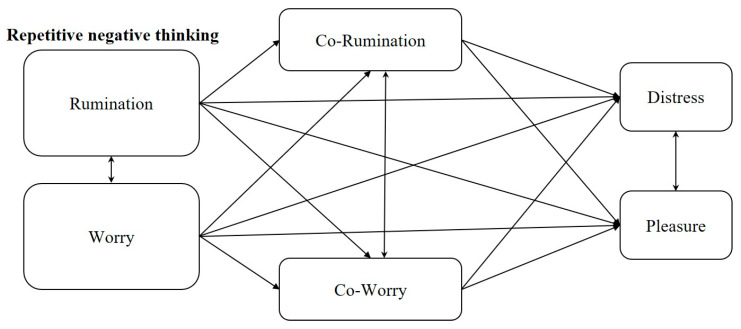
Conceptual mediation model of the relationships between repetitive negative thinking and sexual distress and sexual pleasure.

Considering that sexual distress and sexual pleasure (like sexual satisfaction) may represent opposite sides of the same continuum [31], and that worry and rumination are associated with lower levels of well-being and life satisfaction, the decision was taken to include sexual pleasure as an outcome in the conceptual model (Figure 1). This was to help clarify the relationship between the transdiagnostic factors under study (worry and co-rumination) with two distinct, but complementary, indicators of sexual health.

Considering previous studies in the field of perseverative cognitions [32], we formed the following hypotheses:

**Hypothesis 1** **(H1).** *Worry and rumination are significantly positively correlated.*

Considering previous studies in the field of sexual distress and positive sexual outcomes [31], we expect that:

**Hypothesis 2** **(H2).** *Sexual distress and sexual pleasure are significantly negatively correlated.*

Due to the absence of previous studies in the field of transdiagnostic factors and sexual distress and pleasure, we formed no hypothesis regarding the associations between these variables. However, guided by theory and empirical data concerning a transdiagnostic approach to distress, we sought to answer the following exploratory questions:

Q1: Are worry and rumination associated with sexual distress and sexual pleasure?

Q2: Do co-worry and co-rumination mediate the association between repetitive negative thinking (worry and rumination) and sexual distress and sexual pleasure?

A complete conceptual map of the research is presented in Figure 1.

## 2. Materials and Methods

This is the first study to use data collected from a more extensive project that aimed to correlate sex-related data from cisgender partnered people in exclusive and committed relationships, and it will be the only study to focus on repetitive negative thinking. Data collection was non-probabilistic, leading to a “convenience sample”, and took place on an appropriate professional platform, shared online and disseminated through social networks. To be eligible to complete the questionnaire, participants had to fulfill the following criteria: being 18 years of age or older; being involved in a monogamous relationship; having no medical condition or illness; not taking psychiatric medication; having no diagnosed mental illness; speaking Portuguese as a native language. Before starting the questionnaire, the participants were required to read an informed consent document. This document contained information about the research team, the confidentiality and anonymity of the responses and the lack of monetary compensation for taking part. The participants took an average of 30 min to complete the survey and the drop-out rate (defined as the number of people who read the informed consent document and gave informed consent, but who did not complete the questionnaire) was 47%. This study received approval from the institution’s ethics board and the questionnaire remained available to be answered online from April to July 2019, inclusive (four months). Authorisation was given by all authors of the original versions of the measures used, or of the authors of the translations and validations of the measures used in the present study.

### 2.1. Instruments

#### 2.1.1. General Sociodemographic Questionnaire

Various sociodemographic data were collected, including age, gender, sexual orientation, relationship status, duration of the relationship, educational background and area of residence.

#### 2.1.2. Ruminative Responses Scale (RRQ-10)

The Ruminative Responses Scale (RRQ-10) [33] was developed to measure the frequency of rumination in response to depressive mood. Subjects were asked to reflect on what they usually do, and think, when they are “down”, sad or depressed for each of ten items in a four-point Likert scale ranging from 1 (“almost never”) to 4 (“almost always”). The analysis of the main components of the RRQ-10 identified two factors: “reflection” (adaptive side of rumination) and “brooding” (maladaptive side of rumination). In the present study, a translated and adapted version was used for the Portuguese population and this was developed by Pinto-Gouveia and Dinis (2006). The scale was found to be reliable in the present sample (α = 0.84).

#### 2.1.3. Penn State Worry Questionnaire-Abbreviated (PSWQ-A)

The Penn State Worry Questionnaire-Abbreviated (PSWQ-A) [34] is an abbreviated version of the Penn State Worry Questionnaire (PSWQ) [35], composed of eight of the 16 original items, which was developed to measure worry severity. Items are rated on a five-point Likert scale from 1 (“not at all typical of me”) to 5 (“very typical of me”). Higher scores indicate higher levels of worry. For the current study, the PSWQ-A was adapted from the Portuguese version of PSWQ [36]. The measure showed a high level of reliability when used with the current sample (α = 0.95).

#### 2.1.4. Co-rumination Questionnaire (CRQ)

The Co-rumination Questionnaire (CRQ) [24] is a self-report inventory consisting of 27 items. It uses a five-point Likert scale, which varies between 1 (“not at all true”) and 5 (“completely true”), to measure the extent to which each item describes each participant’s interaction with his or her best friend or close friend. For this study, the scale was adapted for amorous dyads, after authorisation from the author of the original version had been obtained. This scale proved to be reliable (α = 0.96).

#### 2.1.5. Penn State Worry Questionnaire-Abbreviated-Adapted version for Amorous Dyads (Co-PSWQ-A)

As previously mentioned, the Penn State Worry Questionnaire-Abbreviated (PSWQ-A) is a measure consisting of eight items that are used to assess the severity of worrying. Items are rated on a five-point Likert scale from 1 (“not at all typical of me”) to 5 (“very typical of me”), a higher rating corresponding to a higher level of worrying. After obtaining authorisation from the original author, this measure was adapted to apply to amorous dyads in the current study. This scale demonstrated good reliability in this study (α = 0.91).

#### 2.1.6. Female Sexual Distress Scale-Revised (FSDS-R)

The Female Sexual Distress Scale-Revised (FSDS-R) [37] is a 13-item scale that enables the measurement of sexual distress in women with and without sexual dysfunction. It has also been used with samples of males and has proved to be reliable for the evaluation of sexual distress in men [38]. Answers are given using a five-point Likert scale from 0 (“Never”) to 4 (“Always”), referring to the previous 30 days. Total scores may range from 0 to 51, with a high score indicating a high level of sexual distress. The scale proved to be reliable in the present study (α = 0.96).

#### 2.1.7. Sexual Pleasure Scale (SPS)

The Sexual Pleasure Scale (SPS) [39] consists of three items that assess the extent of sexual pleasure obtained, considering three aspects of intimate relationships: sexual activity, sexual relationships and sexual intimacy, on a Likert scale ranging from 1 (“not pleasurable at all”) to 7 (“very pleasurable”). The higher the sum of the three items, which can range from 3 to 21, the higher the level of sexual pleasure. The internal consistency value obtained in this study suggested good reliability (α = 0.98).

### 2.2. Data Analyses

**Figure 2:**
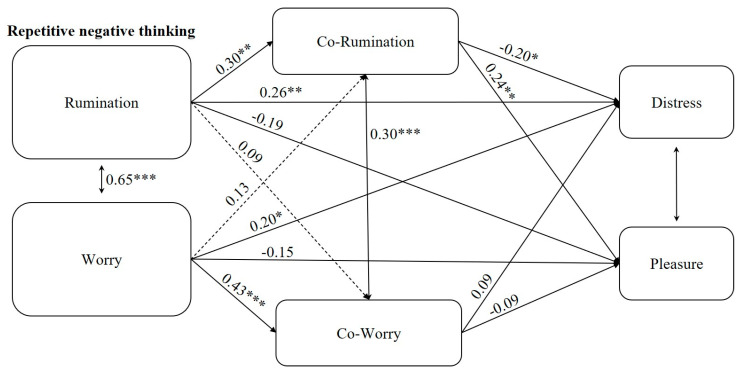
Results of the association of repetitive negative thinking with sexual distress and sexual pleasure. Note: The model is saturated, with no adjustment index. Only the indirect effect for pleasure through co-rumination is significant: Rumination > Co-rumination > Pleasure (Estimate = 0.07; *SE* = 0.03; *p* = 0.029). *** *p* < 0.001. ** *p* < 0.01. * *p* < 0.05. The estimates are positioned above the line they refer to.

First, preliminary analyses were performed to extract descriptive statistics (means and standard deviations) among the variables under study. Next, our hypothesized model (see Figure 2, which includes covariances and regression coefficients) was tested using path analysis with parallel mediation.

Due to the small sample size used, path analysis was selected to explore the multivariate nature of the theoretical assumptions, instead of a full structural latent model [40]. Also, global fit adjustment tests were not applied in the current path analysis, due to its just-identified nature, which means the model presented many freely estimated parameters as observations in the data set [41]. Using the model, estimated perseverative cognitions (rumination and worry) were used as predictors, co-rumination and co-worry were used as mediators and pleasure and distress were used as outcome variables. Analyses were performed using Maximum Likelihood (ML) as the estimator. Indirect effects, using 95% bias-corrected bootstrap confidence intervals based on 1000 samples [42], were estimated. For indirect effects, significance was indicated whenever intervals exceeded 0. For all other effects, significance was considered to be indicated by *p*-values under 0.05. The following linear modelling assumptions were checked: the normal distribution of standardised residuals using Q–Q plots suggested that normality was reasonable, multicollinearity, according to variance inflation factor (VIF) values, ranged from 1.22 to 2.01 < 5 [43] and influential cases using Cook’s distance values ranged from 0.00 to 0.09 < 1 [44]. Analyses were computed using SPSS (v.26, SPSS Inc., Chicago, IL, USA), with structural equation modelling being performed using lavaan [45] designed for R environment [46].

## 3. Results

A total of 208 individuals from a community sample participated in this study; they were aged between 18 and 67 years, with an average of 35.34 years (SD = 10.029). The majority of participants lived in a cohabitation arrangement (68.3%); 72 lived as part of a civil union (34.6%), 70 were married (33.7%) and 60 were single but in a committed relationship (28.8%). The average duration of the couples’ relationship was 8.54 years (SD = 7.490), with a minimum duration of six months and a maximum duration of 40 years. Regarding education level, more than half of the sample held an academic degree (71.2%), with most participants living in a predominantly urban environment (47.6%) (Table 1).

Descriptive statistics are reported in Table 2. Regarding the hypothesised model of parallel mediation, Figure 2 presents standardised coefficients. Rumination significantly predicted distress and pleasure, while worry only predicted distress. Such results suggest that higher levels of rumination are associated with higher values of distress and lower values of pleasure. Similarly, higher levels of worry were related to higher values of distress. The indirect path from rumination via co-rumination to pleasure was statistically significant (*B* = 0.06, SE = 0.03, β = 0.07, *p* = 0.029, 95% CI [0.01, 0.12]), suggesting higher values of pleasure through co-rumination. Other indirect effects were not significant.

## 4. Discussion

The aim of the study was to develop a preliminary cross-sectional study to test whether well-known transdiagnostic explanatory factors (worry and rumination) of emotional distress could also account for sexual distress and its counterpart, pleasure. Considering that most sexual activity occurs in a dyadic context, we tested a mediation model that hypothesised that co-worry and co-rumination may play a mediation role between worry/rumination and sexual pleasure/distress.

The distribution data concerning the variables show some variability in worry, co-worry, rumination and co-rumination in the sample, meaning that the sample of respondents was neither particularly worried nor particularly ruminative, an expected result considering that a clinical sample was not used.

According to the associations between the variables studied (Figure 2), the research hypotheses (H1 and H2) were accepted. Worry and rumination showed strongly significant positive covariation (H1), confirming them to be related, but still independent, constructs [47,48], even using a community sample. A significant positive association was found between co-worry and co-rumination. No previous empirical research that could account for this association was found. Based on theory and the conceptualisation that underlies these two types of repetitive thinking, this result is unsurprising. These behaviours may differ, but each one is an interpersonal strategy to regulate negative emotional states of worry and rumination [27,49]. While rumination and worry are partially overlapping concepts, their interpersonal counterparts (i.e., co-rumination and co-worry) may both serve the purpose of interpersonal emotion regulation (which explains their association). However, they may be expressed through, and associated with, different behaviours and outcomes among romantic dyads. Because, to the best of the authors’ knowledge, this is the first study to examine the relationship between these concepts, how they overlap and differ still needs to be explored.

Nevertheless, it is possible to suggest interpretations that might be clarified through subsequent research. Research with friendship dyads shows that co-rumination may increase a sense of connectedness and proximity among dyads, along with feelings of social support that have a positive impact on the evaluation of the quality of the relationship and the experience of positive emotions related to the relationship [24,50]. Our results seem to suggest that in amorous dyads this effect may lead to higher experience of sexual pleasure.

Some of the characteristics of worry, an underlying process of co-worry, can have an upside in the form of adaptive behaviour. It is possible that, like co-rumination, co-worry serves the purpose of seeking interpersonal emotional regulation, and can also be a precursor to specific adaptive dyadic behaviours, such as seeking help, finding a solution and preventing a problem from happening again. The ways in which co-rumination and co-worry may be related, and the ways in which they may affect romantic dyads, are a rich, yet unexplored, area of research that can be explored in future studies.

As for the association between sexual pleasure and sexual distress, the hypothesis that these are strongly associated dimensions was accepted. In line with the survey’s results, previous research studies have explored the association between sexual distress and sexual satisfaction (a positive outcome of sexual activity linked to sexual satisfaction in different samples) [31,51,52]. However, to the best of the authors’ knowledge, the current study is the first to explore the association between sexual pleasure and sexual distress, placing sexual pleasure, an important sexual right [39], at the heart of research on the sexual outcomes of sexual activity. The results of the current study confirm the hypothesised close relationship between these two constructs, while also confirming their independence. The study provides empirical evidence that the assessment of negative sexual outcomes (such as distress) should be balanced by inclusion of meaningful positive outcomes. This will enable researchers to better understand whether the mechanisms and processes responsible for negative outcomes are different from those related to positive outcomes. This complementary information may help to clarify the complexity of sexual responses in clinical, as well as in non-clinical, samples, contributing to improved practice in health promotion and intervention.

Based on existing explanatory models of psychological distress, the main goal of this preliminary exploratory study was to investigate the development of a mediation model of sexual distress. The aim of the study was to investigate whether a specific set of transdiagnostic processes known as repetitive negative thinking, or perseverative cognitions (worry and rumination) and their interpersonal counterparts (co-worry and co-rumination), could explain the experience of sexual distress.

The results show that both worry and rumination have a significant direct effect on sexual distress, and this is consistent with previous research that has highlighted the role that cognitive factors have on sexual dysfunction. There is an existing consistent body of research that demonstrates that a cognitive-emotional approach such as that proposed by Nobre is valid for understanding sexual dysfunction [53], but also sexual function in community samples [54]. Nobre’s cognitive model of sexual dysfunction has established that cognitive factors, such as sexual beliefs about sexual functioning [55], play an essential role in understanding sexual function. Consideration of the content of sexual beliefs is important for understanding their role and relation to sexual functioning. However, the determining aspect of this relation is the individual’s level of agreement with these beliefs. It is the high level of agreement with dogmatic statements about sexuality or sexual function that is associated with a poorer sexual function, which suggests that there are underlying second-order processes (e.g., inflexibility) which explain sexual functioning.

Existing studies using Nobre’s cognitive-emotional model have used outcome measures of sexual function (e.g., International Index of Erectile Function (IIEF) and Female Sexual Function Index (FSFI)) [56] that do not address the experience of sexual distress. The novelty of the current study is that it moves beyond studying sexual function alone and addresses sexual distress as a core cross-diagnostic feature of sexual dysfunction. This study questioned whether sexual distress could be better explained by well-known explanatory cognitive transdiagnostic processes of emotional distress, such as worry and rumination. The results suggest that this is possible, and add to Nobre’s cognitive-emotional model by identifying patterns of thinking related to the experience of sexual distress associated with sexual functioning. Future studies might evaluate how these patterns of thinking relate to levels of agreement with sexual beliefs in order to contribute to a more comprehensive approach to sexual dysfunction.

It was expected that co-worry and co-rumination would mediate the associations among repetitive negative thinking and sexual distress, but this was not found in the current study. However, an indirect effect of co-rumination on sexual pleasure was observed. Considering that the sample of respondents used was not a clinical sample, this result may be explained by the well-documented positive effect of co-rumination on relationship closeness and feelings of social support [57]. It is possible that co-rumination in amorous dyads serves the purpose of interpersonal emotional regulation and has a positive effect on perceptions of closeness and proximity, contributing to a better sense of relationship well-being. This may create a positive disposition toward sexual activity, which translates into a more pleasurable experience. It is also possible that this effect is due to co-rumination’s association with other variables, such as self-disclosure and sexual communication [58,59], that may contribute to positive sexual outcomes. This clarification is needed from future studies in this field.

Overall, the results suggest that perseverative cognitions, also known as repetitive negative thinking increase sexual distress, a finding consistent with a transdiagnostic approach to emotional and psychological distress. It was also found that the interpersonal counterparts to these behaviours seemed not to have an impact on these associations. However, the finding that co-rumination may mitigate the negative impact of rumination on sexual pleasure is promising. Co-rumination might be linked to sexual communication. Therefore, in a clinical setting, the enactment and maximisation of communication to improve pleasure may be a legitimate goal in improving people’s sexual well-being. This approach, i.e., promoting sexual pleasure and sexual well-being, can complement other important therapeutic goals, such as improving sexual function and/or diminishing sexual distress.

### Limits

This preliminary study is not free of limitations. Some of them are shared by most sex research studies (e.g., the self-selection bias; sample size). Specifically, the study sample mostly comprised female participants with a high level of education, mostly living in urban/suburban areas. Thus, the study’s results should be interpreted with caution, taking this element into consideration.

Although relevant clinical models can be tested using community samples, the current study is clearly limited by the absence of a clinical sample to clarify the associations found and enhance the clinical significance of the study. This is a cross-sectional study, and, as such, the associations presented among variables are based on theory, and a clear direction on the association between variables, as well as causality, cannot be determined. Nevertheless, this preliminary study is important, given the lack of studies in this field. The study has been developed in line with Salthouse’s claim that “we should also resist universal rejection of analytical procedures that can be informative when their limitations are recognized” [60] (p. 797), since we believe that it can open a new area of research and inform future research in this field.

Furthermore, the measure of sexual distress used in the study is, according to some authors, insufficient to grasp the complexities of the concept of sexual distress and this might limit the coverage of the research [61].

Finally, due to the theoretical framing of the study as being dyadic, and the complexity that the inclusion of different relationship structures would have brought to the data analysis, people in non-committed relationships and people involved in consensual non-monogamic relationships were excluded from the research.

## 5. Conclusions

This innovative study aimed to establish whether transdiagnostic factors characterised by repetitive negative thinking (worry and rumination) were associated with sexual distress and its counterpart, sexual pleasure, using a mediation model that used co-worry and co-rumination as mediators. The results clearly demonstrate that repetitive negative thinking is linked to both sexual pleasure and sexual distress, opening a new field of research with implications for both theory and practice. In terms of theory, the results enable an expansion of existing cognitive models of sexual dysfunction and contribute to existing knowledge by demonstrating how transdiagnostic factors might be used in a clinical approach to sexual distress, a claim which might be tested using clinical samples in the context of sexual dysfunction. Furthermore, the results demonstrate that repetitive negative thinking is also negatively associated with positive experiences related to sexual activity, such as sexual pleasure. To the authors’ knowledge, this is the first study to demonstrate that cognitive factors have an association with sexual pleasure (not orgasm). The possibility that this association may be minimized through interpersonal factors (co-rumination) is a promising field to be explored in future research, particularly in clinical settings.

## Figures and Tables

**Table 1:** Sociodemographic characteristics of participants (*n* = 208).

Characteristics	*N*	%
Age (mean ± SD)	35.34 ± 10.029	-
Min	18	-
Max	67	-
Gender	-	-
Female	160	76.9
Male	46	22.1
Non-binary	1	0.5
Agender	1	0.5
Transgender ^a^	-	-
Yes	11	5.3
No	194	93.3
Don’t know	2	1.0
Sexual orientation	-	-
Heterosexual	185	88.9
Gay	1	0.5
Lesbian	7	3.4
Bisexual	11	5.3
Undefined	1	0.5
Other	3	1.4
Relationship duration (mean ± SD)	8.54 ± 7.490 (years)	-
Min	6 (months)	-
Max	40 (years)	-
Relationship status ^a^	-	-
Civil union/cohabitation	72	34.6
Marriage	70	33.7
Single in a committed relationship	60	28.8
Other	5	2.4
Educational level	-	-
Middle school	2	1.0
High school	52	25.0
Bachelor’s degree	82	39.4
Master’s degree	54	26.0
Doctoral degree	12	5.8
Other	6	2.9
Area of residence ^a^	-	-
Urban area	99	47.6
Suburban area	70	33.7
Urbanised centre of a rural area	14	6.7
Rural area next to an urban area	13	6.3
Rural area with high population density	5	2.4
Rural area with little population density	4	1.9

^a^ Ns do not sum to the total, due to missing values: transgender (1), marital and relationship status (1), area of residence (3).

**Table 2:** Descriptive statistics for variables in the study (*n* = 208).

Variable	*M*(*SD*) {min–max}
1. Rumination	19.50 (5.70) {9–34}
2. Worry	22.29 (9.12) {8–40}
3. Co-rumination	66.31 (21.02) {29–128}
4. Co-worry	20.44 (7.02) {8–38}
5. Sexual Pleasure	18.35 (4.73) {3–21}
6. Sexual Distress	12.71 (13.74) {0–52}

Note: Mean (*M*), standard deviation (*SD*), range values {min–max}. 1 and 2 are predictors, 3 and 4 are mediators, 5 and 6 are outcomes. *** *p* < 0.001; ** *p* < 0.01; * *p* < 0.05.

## References

[1] Griffiths N. (2017). The Relationship between Co-Rumination and Depression, Anxiety and Social Anxiety in a UK Community Sample. Ph.D. Thesis.

[2] Sauer-Zavala S., Gutner C.A., Farchione T.J., Boettcher H.T., Bullis J.R., Barlow D.H. (2017). Current definitions of “transdiagnostic” in treatment development: A search for consensus. Behaviour.

[3] Wahl K., Ehring T., Kley H., Lieb R., Meyer A., Kordon A., Heinzel C.V., Mazanec M., Schönfeld S. (2019). Is repetitive negative thinking a transdiagnostic process? A comparison of key processes of RNT in depression, generalized anxiety disorder, obsessive-compulsive disorder, and community controls. J. Behav. Exp. Psychiatry.

[4] Shifren J.L., Monz B.U., Russo P.A., Segreti A., Johannes C.B. (2008). Sexual problems and distress in united states women: Prevalence and correlates. Obs. Gynecol.

[5] Forbes M.K., Baillie A.J., Schniering C.A. (2016). A structural equation modeling analysis of the relationships between depression, anxiety, and sexual problems over time. J. Sex. Res..

[6] Borkovec T.D., Davey G.C.L., Tallis F. (1994). The nature, functions, and origins of worry. Worrying: Perspectives on Theory, Assessment, and Treatment.

[7] Sweeny K., Dooley M.D. (2017). The surprising upsides of worry. Soc. Personal Psychol. Compass..

[8] Brown T.A., Tung E.S. (2018). The contribution of worry behaviors to the diagnosis of generalized anxiety disorder. J. Psychopathol. Behav. Assess..

[9] Gralla O., Knoll N., Fenske S., Spivak I., Hoffmann M., Rönnebeck C., Lenk S., Hoschke B., May M. (2008). Worry, desire, and sexual satisfaction and their association with severity of ED and age. J. Sex. Med..

[10] Katz R.C., Jardine D. (1999). The relationship between worry, sexual aversion, and low sexual desire. J. Sex. Marital..

[11] Nolen-Hoeksema S., Wisco B.E., Lyubomirsky S. (2008). Rethinking rumination. Perspect. Psychol. Sci..

[12] McLaughlin K.A., Nolen-Hoeksema S. (2011). Rumination as a transdiagnostic factor in depression and anxiety. Behav. Res..

[13] Borders A., Guillén L.A., Meyer I.H. (2014). Rumination, sexual orientation uncertainty, and psychological distress in sexual minority university students. Couns. Psychol..

[14] Conway M., Mendelson M., Giannopoulos C., Csank P., Holm S. (2004). Childhood and adult sexual abuse, rumination on sadness, and dysphoria. Child. Abus. Negl..

[15] McCool-Myers M., Theurich M., Zuelke A., Knuettel H., Apfelbacher C. (2018). Predictors of female sexual dysfunction: A systematic review and qualitative analysis through gender inequality paradigms. BMC Womens Health.

[16] Rowland D.L., Kolba T.N. (2018). The burden of sexual problems: Perceived effects on men’s and women’s sexual partners. J. Sex. Res..

[17] Rosen R.C., Shifren J.L., Monz B.U., Odom D.M., Russo P.A., Johannes C.B. (2009). Correlates of sexually related personal distress in women with low sexual desire. J. Sex. Med..

[18] Nimbi F.M., Tripodi F., Rossi R., Navarro-Cremades F., Simonelli C. (2020). Male sexual desire: An overview of biological, psychological, sexual, relational, and cultural factors influencing desire. Sex. Med. Rev..

[19] Hevesi K., Gergely H.B., Kolba T., Rowland D. (2020). Self-reported reasons for having difficulty reaching orgasm during partnered sex: Relation to orgasmic pleasure. J. Psychosom. Obs. Gynecol..

[20] Hendrickx L., Gijs L., Enzlin P. (2019). Who’s distressed by sexual difficulties? Exploring associations between personal, perceived partner, and relational distress and sexual difficulties in heterosexual men and women. J. Sex. Res..

[21] Calmes C.A., Roberts J.E. (2008). Rumination in interpersonal relationships: Does co-rumination explain gender differences in emotional distress and relationship satisfaction among college students?. Cogn. Res..

[22] Spendelow J.S., Simonds L.M., Avery R.E. (2017). The relationship between co-rumination and internalizing problems: A systematic review and meta-analysis. Clin. Psychol. Psychother..

[23] Dombrowski C. (2014). Gender Differences in Co-Rumination, Co-Worry, and Internalizing Symptoms in Late Adolescence. Master’s Thesis.

[24] Rose A. (2002). Co–rumination in the friendships of girls and boys. Child. Dev..

[25] Borkovec T.D., Ray W.J., Stober J. (1998). Worry: A cognitive phenomenon intimately inked to affective, physiological, and interpersonal behavioral processes. Cognit. Ther. Res..

[26] Parkinson B., Simons G. (2012). Worry spreads: Interpersonal transfer of problem-related anxiety. Cogn. Emot..

[27] Parkinson B., Simons G., Niven K. (2016). Sharing concerns: Interpersonal worry regulation in romantic couples. Emotion.

[28] Goldstein M. (2014). Co-Worry in Friendship Dyads. Honors Scholar Thesis.

[29] Weiss B.J., Hope D.A. (2011). A preliminary investigation of worry content in sexual minorities. J. Anxiety Disord..

[30] Calzada E.J., Brown E.J., Doyle M.E. (2011). Psychiatric symptoms as a predictor of sexual aggression among male college students. J. Aggress. Maltreatment Trauma.

[31] Stephenson K.R., Meston C.M. (2010). Differentiating components of sexual well-being in women: Are sexual satisfaction and sexual distress independent constructs?. J. Sex. Med..

[32] Topper M., Emmelkamp P., Watkins E., Ehring T. (2014). Development and assessment of brief versions of the Penn State Worry Questionnaire and the Ruminative Response Scale. Br. J. Clin. Psychol..

[33] Treynor W., Gonzalez R. (2003). Rumination reconsidered: A psychometric analysis. Cogn. Res..

[34] Hopko D., Stanley M., Reas D. (2003). Assessing worry in older adults: Confirmatory factor analysis of the Penn State Worry Questionnaire and psychometric properties of an abbreviated model. Psychol. Assess..

[35] Meyer T.J., Miller M.L., Metzger R.L., Borkovec T. (1990). Development and validation of the Penn State Worry Questionnaire. Behav. Res. Ther..

[36] Jiménez-Ros A.M., Carmona-Márquez J., Pascual L.M. (2019). Pathological worry in portugal: The portuguese version of the Penn State Worry Questionnaire (PSWQ). Span. J. Psychol..

[37] Derogatis L., Clayton A., Lewis-D’Agostino D., Wunderlich G., Fu Y. (2008). Validation of the Female Sexual Distress Scale-Revised for assessing distress in women with hypoactive sexual desire disorder. J. Sex. Med..

[38] Santos-Iglesias P., Mohamed B., Danko A., Walker L.M. (2018). Psychometric validation of the female sexual distress scale in male samples. Arch. Sex. Behav..

[39] Pascoal P.M., Sanchez D.T., Raposo C.F., Pechorro P. (2016). Initial validation of the sexual pleasure scale in clinical and non-clinical samples of partnered heterosexual people. J. Sex. Med..

[40] Glazer S., Beehr T.A. (2005). Consistency of implications of three role Stressors across four countries. J. Organ. Behav..

[41] Goodboy A.K., Kline R.B. (2017). Statistical and practical concerns with published communication research featuring structural equation modeling. Commun. Res. Rep..

[42] Hayes A.F. (2013). Introduction to Mediation, Moderation, and Conditional Process Analysis.

[43] Hair J.F., Black W.C., Babin B.J., Anderson R.E. (2010). Multivariate Data Analysis.

[44] Robinson A., Cook R.D., Weisberg S. (1984). Residuals and influence in regression. J. R Stat. Soc. Ser. A.

[45] Rosseel Y. (2012). Lavaan: An R package for structural equation modeling. J. Stat. Softw..

[46] (2020). R Core Team R: A Language and Environment for Statistical Computing. http://www.R-project.org/.

[47] Szasz T. (1991). The medicalization of sex. J. Humanist. Psychol..

[48] Fresco D.M., Frankel A.N., Mennin D.S., Turk C.L., Heimberg R.G. (2002). Distinct and overlapping features of rumination and worry: The relationship of cognitive production to negative affective states. Cogn. Res..

[49] Hofmann S.G. (2014). Interpersonal emotion regulation model of mood and anxiety disorders. Cogn. Res..

[50] Smith R.L., Rose A.J. (2011). The “cost of caring” in youths’ friendships: Considering associations among social perspective taking, co-rumination, and empathetic distress. Dev. Psychol..

[51] Pascoal P.M., Narciso I.S., Pereira N.M. (2013). What is sexual satisfaction? Thematic analysis of lay people’s definitions. J. Sex. Res..

[52] Pascoal P.M., Shaughnessy K., Almeida M.J. (2019). A thematic analysis of a sample of partnered lesbian, gay, and bisexual people’s concepts of sexual satisfaction. Psychol. Sex..

[53] Nobre P., Pinto-Gouveia J. (2006). Dysfunctional sexual beliefs as vulnerability factors for sexual dysfunction. J. Sex. Res..

[54] Nobre P., Pinto-Gouveia J. (2006). Emotions during sexual activity: Differences between sexually functional and dysfunctional men and women. Arch. Sex. Behav..

[55] Pascoal P.M., Rosa P.J., da Silva E.P., Nobre P.J. (2018). Sexual beliefs and sexual functioning: The mediating role of cognitive distraction. Int. J. Sex. Heal..

[56] Nobre P., Pinto-Gouveia J., Gomes F. (2006). Prevalence and comorbidity of sexual dysfunctions in a portuguese clinical sample. J. Sex. Marital.

[57] Rose A.J., Carlson W., Waller E.M. (2007). Prospective associations of co-rumination with friendship and emotional adjustment: Considering the socioemotional trade-offs of co-rumination. Dev. Psychol..

[58] Pascoal P.M., Lopes C.R., Rosa P.J. (2019). The mediating role of sexual self-disclosure satisfaction in the association between expression of feelings and sexual satisfaction in heterosexual adults. [O papel mediador da autorrevelação sexual na relação entre a expressão de sentimentos e a satisfaç. Rev. Lat. Psicol..

[59] Roels R., Janssen E. (2020). Sexual and relationship satisfaction in young, heterosexual couples: The role of sexual frequency and sexual communication. J. Sex. Med..

[60] Salthouse T.A. (2011). All data collection and analysis methods have limitations: Reply to Rabbitt (2011) and Raz and Lindenberger. Psychol. Bull..

[61] Hendrickx L., Gijs L., Janssen E., Enzlin P. (2016). Predictors of sexual distress in women with desire and arousal difficulties: Distinguishing between personal, partner, and interpersonal distress. J. Sex. Med..

